# Right anterolateral thoracotomy: an attractive alternative to repeat sternotomy for high-risk patients undergoing reoperative mitral and tricuspid valve surgery

**DOI:** 10.1186/s13019-017-0645-x

**Published:** 2017-09-21

**Authors:** Hailong Cao, Qing Zhou, Fudong Fan, Yunxing Xue, Jun Pan, Dongjin Wang

**Affiliations:** Department of Thoracic and Cardiovascular Surgery, the Affiliated Drum Tower Hospital of Nanjing, University Medical School, 321 Zhongshan RD, Nanjing, 210008 China

**Keywords:** Right anterolateral thoracotomy, Reoperation, Mitral and tricuspid valve surgery, High-risk

## Abstract

**Background:**

Reoperative cardiac valve surgery via sternotomy is associated with a substantial morbidity and mortality. This study evaluated the right anterolateral thoracotomy for high-risk patients undergoing mitral and tricuspid valve redo procedures.

**Methods:**

Out of a series of 173 patients undergoing redo cardiac valve surgery, 24 patients were reoperative via the right anterolateral thoracotomy as the high-risk group on the basis of the proximity of the heart and great vessels to the sternum and the presence and location of patent bypass grafts.

**Results:**

In all cases, sternotomy was avoided. The mitral valve and tricuspid valve were replaced in 4 and 19 patients and repaired in 1 and 2 patients, respectively. Moreover, left atrial folding was performed in 5 patients. Mortality was 8.3%. All other patients had uneventful outcomes and normal valve function at follow-up.

**Conclusions:**

Reoperative cardiac valve surgery can be performed safely using the right anterolateral thoracotomy in high-risk patients. It offers enough exposure. It minimizes the need for cardiac dissection, and thus, the risk for injury. Avoiding a high-risk resternotomy increases patients comfort and safety of redo mitral and tricuspid valve surgery.

**Electronic supplementary material:**

The online version of this article (10.1186/s13019-017-0645-x) contains supplementary material, which is available to authorized users.

## Background

Reoperative cardiac valve surgery through a median sternotomy continues to be a common surgical approach but is technically challenging. It has several associated risks including injury to the right ventricle, injury to patent coronary artery bypass grafts and bleeding, thereby increasing operative morbidity and mortality [1]. In the setting of reoperative cardiac surgery, the redo-sternotomy had been proven to be one of the most dangerous phases of the operation, particularly for patients with huge heart or firm and gapless adhesion [1, 2].

Several protective strategies have been described for reoperative cardiac valve surgical procedures, including femoral vessel exposure before sternotomy [3], prophylactic initiation of cardiopulmonary bypass [1], and a right thoracotomy approach [4–6]. Rountine computed tomography scanning is performed to visualize the relationship of the mediastinal contents to the sternum and to identify the patients at risk for injury during reentry [7]. However, it still cannot rule out accidental injury during sternotomy [3]. Moreover, potential postoperative complications, such as mediastinitis, sternal dehiscence, and phrenic nerve injury, have been reported [8]. Therefore, we herein present our experience that reoperative mitral and tricuspid valve surgery can be performed safely using the right anterolateral thoracotomy in high-risk patients.

## Methods

### Patient enrollment

From December 2012 to July 2016, 173 patients underwent redo cardiac valve surgery at department of thoracic and cardiovascular surgery in the Affiliated Drum Tower Hospital of Nanjing University Medical School. All of these patients had at least one prior operation that had been performed via a median sternotomy. All included patients had given written informed consent for their detail clinical data. Twenty four high-risk patients were chosen the right anterolateral thoracotomy, others were reoperative via the primary median sternotomy. The study was conducted according to the Helsinki Declaration and approved by the ethics committee of Nanjing University.

### Definition of high-risk patients

**Fig. 1:**
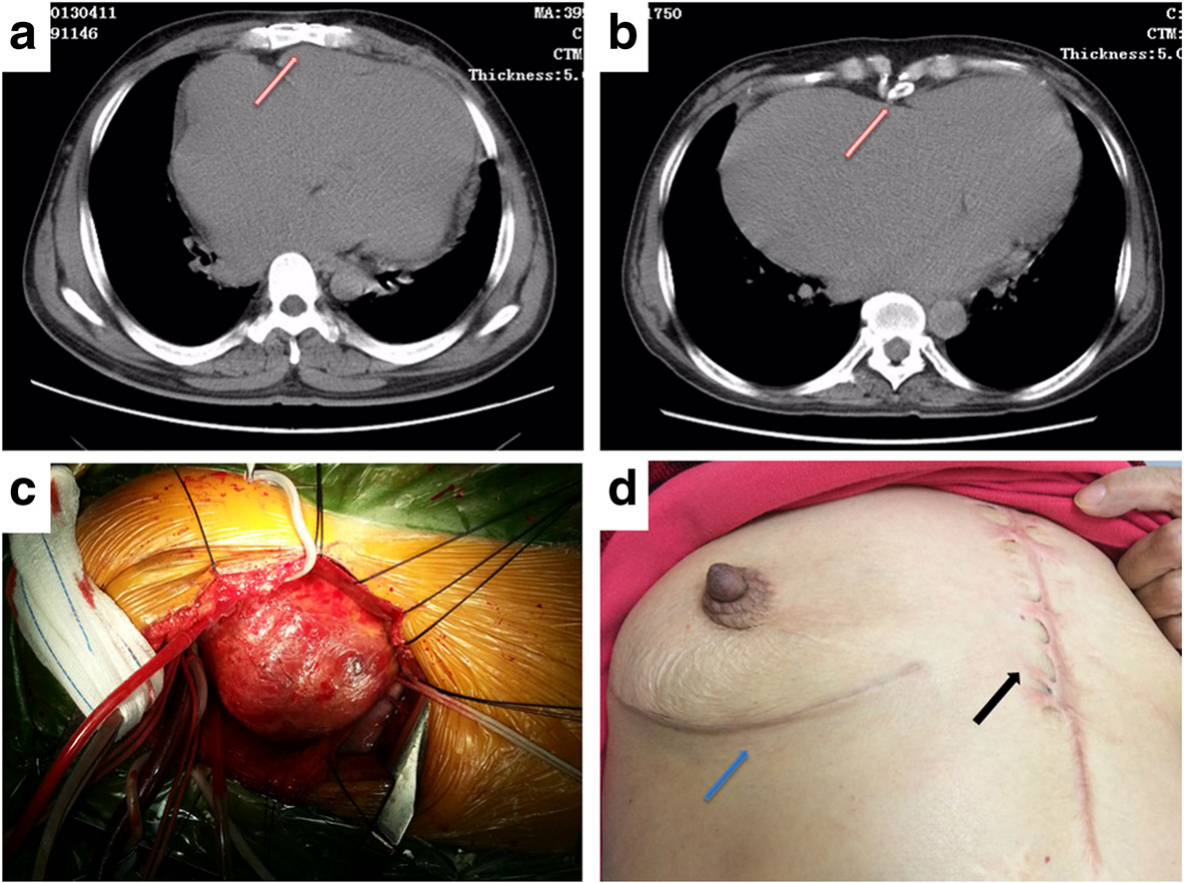
**a** CT scan shows the firm adhesion between the right ventricle and the sternum in high-risk patients; **b** CT scan shows the firm adhesion between the right atrium and the sternum in high-risk patients; **c** The exposure by the right anterolateral thoracotomy after beginning of cardiopulmonary bypass; **d** The primary incision (black arrow) and the redo incision (blue arrow)

(1) proximity (<5 mm) of right atrium or ventricle to the sternum (Fig. 1a, b); (2) previously placed bypass graft crossing midline with <1 cm distance from the posterior surface of the sternum, or fixed to the sternum (lack of movement on angiography); (3) proximity of ascending aorta to the sternum (<5 mm); (4) history of mediastinitis, >2 sternotomies, chest radiation; (5) severe pulmonary hypertension, severe dilated right ventricle; (6) reoperation within 6 months from the last operation [2].

### Operation technique

Under general anesthesia with a single or dual lumen endotracheal tube, the patients were positioned in a 30° anterior oblique positionafter the fourth rib has been marked anteriorly. External defibrillation pads were placed. After the right anterolateral thoracotomy was performed through the right fourth intercostalspace via an approximate 12 cm incision (Fig. 1c), cardiopulmonary bypass was initiated using cannulation through right femoral artery, right femoral vein and superior vena cava under transesophageal echocardiography guidance. In case of pleural adhesions due to prior surgery, the right lung had to be dissected from the pericardium. The operative field was filled with carbon dioxide gas at the rate of 5 L/min throughout the surgery. After beginning cardiopulmonary bypass, dissection of the ascending aorta for conventional aortic cross-clamping was initially attempted in all patients. Twenty two patients underwent cardiopulmonary bypass with a mild hypothermia (32 °C to 34 °C), antegrade cold blood high potassium cardioplegic arrest. Two patients were cooled to 24 °C and induced ventricular fibrillation to perform the surgery under continued retrograde perfusion via coronary sinus for failure to dissect the ascending aorta.

### Statistical analysis

Statistical analysis was performed using SPSS, version 15.0 (SPSS, Chicago, IL). Continuous variables are expressed as mean ± SD. Categoric variables are presented as number and proportions.

## Results

### Patient characteristics

The demographic data of the patients are shown in Table 1. We studied 24 patients from this series (11 females, 13 males, mean age 51.3 ± 8.6 years) who underwent redo mitral and tricuspid valve surgery via right anterolateral thoracotomy. Two patients had already undergone two previous cardiac operations. Table 1 lists all primary operations. Five and Twenty one patients had mitral and tricuspid valve insufficiency, respectively. Twenty two patients had atrial fibrillation. Fifteen patients were in New York Heart Association Class III, and left 9 were in Class IV. Mean left ventricular ejection fraction was 47.5% and mean cardiothoracic ratio was 69%. Left and right atrial diameter were 79 ± 3.1 and 72 ± 25, respectively.

**Table 1:** Patients’ demographic and preoperative clinical data

Variable	Mean ± SD or Number (%)
Age(years)	51.3 ± 8.6
Gender (n)	
Male	13 (54.2%)
Female	11 (45.8%)
Perivous operation time (n)	
1	22 (91.7%)
2	2 (8.3%)
Primary operation (n)	
Mitral valve repair	2
Mitral valve replacement	18
Aortic valve replacement	7
Atrial septal defect repair	3
Tricuspid valvuloplasty	9
Coronary artery bypass grafting	3
New York Heart Association class (n)	
Class III	15 (62.5%)
Class IV	9 (37.5%)
Mitral valve insufficiency (n)	5
Tricuspid valve insufficiency (n)	21
Atrial fibrillation (n)	22
Left ventricular ejection fraction (%)	47.5 ± 13.2
Left atrial diameter (mm)	79 ± 3.1
Right atrial diameter (mm)	72 ± 25
Cardiothoracic ratio (%)	69 ± 18

### Operative characteristics

Table 2 lists all category of operations. Most patients received tricuspid valve replacement. The patients were in the operating room for a mean of 268 min, and had an average duration of cardiopulmonary bypass and cross-clamp of 133 and 67 min, respectively. Moreover, the blood loss during operation was 238 ± 116 ml, and the blood transfusion was 325 ± 246 ml. The incision length was 12.6 ± 2.3 cm (Fig. 1d). The intraoperative course was uneventful and no patient was converted to a full sternotomy.

**Table 2:** Summary of Operative Variables

Variable	Mean ± SD or Number (%)
Category of operation (n)	
Mitral valve repair	1
Mitral valve replacement	4
Tricuspid valvuloplasty	2
Tricuspid valve replacement	19
Left atrial folding	5
Total surgery (min)	268 ± 89
Cardiopulmonary bypass (min)	133 ± 49
Cross-clamp (min)	67 ± 34
Blood loss during operation (ml)	238 ± 116
Blood transfusion during operation (ml)	325 ± 246
Incision length (cm)	12.6 ± 2.3

### Outcomes and follow-up

Postoperative data was shown in Table 3. The chest drainage volume of the first 24 h was 225 ± 87 mL, and there was no postoperative blood transfusion in 17 patients. There were 8 patients whose duration of mechanical ventilation exceeded 24 h and 12 patients whose intensive care unit stay time exceeded 3 days. Six patients received continuous renal replacement therapy for acute renal failure or oliguresis. Extracorporeal membrane oxygenation was performed in 4 patients for low output syndrome (3 patients) and severe hypoxemia. Two patients died for low output syndrome causing multisystem organ failure and lung hemorrhage causing uncontrollablepulmonary infection. The left 22 patients’ postoperative hospital stay was 16.4 ± 7.9 days.

**Table 3:** Postoperative data of all patients

Variable	Mean ± SD or Number (%)
Drainage at the first day (mL)	225 ± 87
Ventilator >24 h (n)	8 (33.3%)
Intensive care unit stay >3 day (n)	12 (50%)
Continuous renal replacement therapy (n)	6 (25%)
Extracorporeal membrane oxygenation (n)	4 (16.7%)
Low output syndrome (n)	3 (12.5%)
Lung hemorrhage (n)	2 (8.3%)
Postoperative hospital stay (days)	16.4 ± 7.9
Mortality (%)	2 (8.3%)

Postdischarge follow-up information was obtained by follow-up clinc and telephone interview. The duration of follow-up ranged 6 to 42 months and follow-up rate was 100%. All the patients were surviving at the time of follow-up and willing to personally provide information regarding their functional status. There were no late deaths or cardiovascular accident during the follow-up.

## Discussion

More and more minimally invasive techniques for cardiac valve surgery have been proven comparable results to conventional techniques [9]. Therefore, there is a greater interest in less invasive approaches to the heart, especially when these alternative access routes decrease the surgical risk and also do not compromise the quality of surgery via the standard approach. Due to these findings, we performed redo mitral and tricuspid valve surgery through a less invasive right anterolateral thoracotomy in high-risk patients. This series documented 24 patients undergoing the less invasive technique for redo cardiac valve procedures.

As a result, the right anterolateral approach offered excellent visualization of the mitral and tricuspid valve structures due to a direct-line view [10]. Median sternotomy for access in reoperations of cardiac valve requires more extensive and time-consuming dissection of adhesions. Reentry via a sternotomy bears the potential risk of direct injury to the right atrium and ventricle and is associated with bleeding complications and blood transfusion requirements [1]. In case of previous coronary artery bypass conduits, venous and especially internal mammary artery grafts (in our series, three patients) are prone to injury during reintervention. Hemorrhage from the heart or great vessels during sternotomy for cardiac reoperations has been reported to occur in 3.6% to 4.3% of cases [2]. Approximately one third of these patients die [2]. Our current data have not reported any major hemorrhage or mortality associated with dissection of adhesions via right thoracotomy. In our group, indeed, which patients with a severe dilated atrium or ventricle or the location of patent bypass grafts, it was believed that this risk was even higher via a sternotomy [11].

However, the dissection of the ascending aorta to achieve aortic cross-clamping is a major concern in patients via the right anterolateral thoracotomy. In the present two cases, dissecting the ascending aorta for aortic cross-clamping was not possible due to severe adhesion or location of patent bypass graft. We therefore decided to apply a strategy involving hypothermic fibrillatory arrest without an aortic cross-clamp, which is known as the no-touch technique [12]. Adequate myocardial protection against both ischemic and distention injuries and reducing the risk of stroke are generally major concerns in left heart surgery performed under fibrillatory arrest [13]. In order to achieve successful myocardial protection, we opened the left atrium immediately upon fibrillation in order to keep the left ventricle decompensated. Moreover, carbon dioxide gas was infused into the operative field to ensure that air did not enter the systemic circulation, and the mean arterial perfusion pressure was maintained at over 30 mmHg in order to keep the aortic valve closed. Transesophageal echocardiography confirmed that no intracardiac air was present before cardioversion.

Moreover, poor exposure of the ventricles requires specific strategies regarding de-airing, pacing-wire insertion, and defibrillation. It is mandatory to allow the left heart to fill with blood before the atrial septum is closed completely. Only the aortic root is de-aired before the aortic cross-clamp is opened. The ventricular pacing wire is inserted on the empty heart during cardiopulmonary bypass. Defibrillation can be performed with preoperatively fixed external paddles.

Isolated reoperative tricuspid valve surgery is considered to be associated with high operative risk [4]. Although the operation may not be technically complicated, the increased risk is usually due to the fact that patients are referred for surgery late in their disease process. Such patients often have evidence of right heart failure and associated complications. It is unknown whether poor postoperative outcome is related to the severity of tricuspid regurgitation itself or to the poor overall status of such patients. In previous studies, hospital mortality ranged from 0% to 37% [14, 15]. However, mortality of our study was lower than that of previous studies, and prognosis of present study was better than that of previous studies. It was convincing that prevention of dissection of the right ventricle, is additionally protective against dilatation of the right ventricle after surgery that would result in poor right heart function. Our policy is to use bioprosthetic valves (Medtronic Hancock II or Carpentier-Edwards Perimount) for tricuspid valve replacement in all patients to avoid excessive anticoagulation, regardless of patient age or presence of a previously implanted mechanical prosthesis in the aortic and/or mitral position.

In this series, we found that a dual lumen endotracheal tube was necessary. There were two cases of pulmonary hemorrhage in our group at the early stage by using a single lumen intubation. It was caused by excessively compressing lung during dissecting adhesions of right atrium and ascending aorta. After that, we used a double lumen endotracheal tube to avoid excessive lung injury. As a result, there was no pulmonary hemorrhag by the double lumen endotracheal tube. Severe pulmonary dysfunction, as determined by the PO2/FiO2 ratio [16], is also a relative contraindication to the right thoracotomy approach. In the present series, two patients had preoperative severe pulmonary dysfunction. One died of lung hemorrhage causing uncontrollable pulmonary infection, another weaned from the ventilator required tracheostomy but recovered fully.

The blood loss and transfusion are denitely less using this approach, probably because of the avoidance of sternotomy. The added advantage of totally eradicating the risk of deep sternal infection is invaluable. Phrenic nerve damage, which is especially attributed to right anterolateral thoracotomy, was not seen in our series. Since the nerve is always easily visible, there should not be incidental damage. Moreover, the intact thorax offers earlier mobilization and return to daily life activities [6].

### Limitations

The current study has some limitations. First, our patient population is small because of the rarity of patients requiring a redo cardiac valve surgery with a high-risk resternotomy. Second, the heterogeneity of this group of patients with regard to demographics, prior surgery, preoperative cardiac function, and co-morbid conditions makes risk adjustment impossible, so we did not do a case–control study between the right anterolateral thoracotomy and the resternotomy. Moreover, we accept that different valve reoperations provide different surgical challenges, the preoperative status of the patient can have a profound influence on the surgical outcome [17].

## Conclusion

The right anterolateral thoracotomy has become a standard approach for redo mitral and tricuspid valve surgery in high-risk patients at our institution. It avoids a high-risk resternotomy, and can be performed safely and reduces the possibility of injury to the heart.

## Additional file

Additional file 1: Original data. (XLSX 54 kb)
